# Can Big Data Be Used to Monitor the Mental Health Consequences of COVID-19?

**DOI:** 10.3389/ijph.2021.633451

**Published:** 2021-04-08

**Authors:** Nicola Julia Aebi, David De Ridder, Carlos Ochoa, Dusan Petrovic, Marta Fadda, Suzanne Elayan, Martin Sykora, Milo Puhan, John A. Naslund, Stephen J. Mooney, Oliver Gruebner

**Affiliations:** ^1^ Swiss Tropical and Public Health Institute, Basel, Switzerland; ^2^ University of Basel, Basel, Switzerland; ^3^ University of Geneva, Faculty of Medicine, Institute of Global Health, Geneva, Switzerland; ^4^ École Polytechnique Fédérale de Lausanne, Lausanne, Switzerland; ^5^ Institute for Environmental Sciences, University of Geneva, Geneva, Switzerland; ^6^ Department of Epidemiology and Health Systems (DESS), University Center for General Medicine and Public Health (UNISANTE), Lausanne, Switzerland; ^7^ Centre for Environment and Health, School of Public Health, Department of Epidemiology and Biostatistics, Imperial College London, London, United Kingdom; ^8^ University of Lugano, Faculty of Biomedical Sciences, Lugano, Switzerland; ^9^ Centre for Information Management, Loughborough University, Leicestershire, United Kingdom; ^10^ University of Zurich, Epidemiology, Biostatistics and Prevention Institute, Zurich, Switzerland; ^11^ Harvard Medical School, Boston, MA, United States; ^12^ University of Washington, Department of Epidemiology, Seattle, WA, United States; ^13^ University of Zurich, Department of Geography, Zurich, Switzerland

**Keywords:** surveillance, digital epidemiology, spatial epidemiology, digital health geography, social media

## Introduction

The COVID-19 pandemic has profound mental health consequences [1]. Yet, opportunities to monitor and mitigate mental health problems in this context remain scarce [2]. At the same time, nearly half of the world’s population (49%) now use social media and digital tools such as natural language processing have improved considerably, particularly for mental health [3]. Using these tools, researchers have identified and monitored signs of mental illness reflected in social media data including stress, loneliness, depression, or post-traumatic stress [4]. Such approaches, part of a growing field called digital epidemiology, could help identify populations in need of mental health support during the current pandemic. More specifically, sentiment analysis of content posted on popular social media platforms, combined with detection of spatiotemporal disease incidence changes could provide decision makers and public health experts with critical information to supplement traditional epidemiological data sources, and to inform the implementation of targeted mental health interventions [5–7].

## Ethical and Legal Concerns of Big Data

Despite the promise of Big Data, it is important to acknowledge that these digital epidemiologic approaches also raise ethical and legal concerns, particularly with regards to consent, privacy expectations, data protection, and security. Social media users posting publicly may not have consented to being in a research study, and those suffering from mental illness may not have intended for their posts to reveal their health status. People may have shared their information via social media while in a temporary vulnerable state of mind, e.g., during a crisis or during a disease outbreak. In this case, they may not necessarily realize that what they share can potentially be collected and analyzed by third parties, either for relief, marketing, or scientific activities. Yet being identified as mentally ill might cause stigma in private life, at work, become a source of discrimination, and might affect access and use of healthcare services. These ethical issues are compounded by potential legal issues, including regulations regarding the security and protection of the data, and the malicious use of sensitive, health-related data by third parties. Therefore, methodologies, such as de-identification and anonymization, can ensure data protection and privacy by removing personal identifiers. Geo-masking or aggregation of spatial data are also applied to remove geographical attributes [8].

## Methodological Concerns of Big Data

Research or interventions based on Big Data are subject to validity concerns. The theory underlying formal statistics typically assumes random sampling [9], but because e.g., social media users may not be representative of the general population in terms of demographics or socioeconomic factors, analyzing these data without accounting for the potential non-representativeness may result in selection bias and low internal and external validity [10]. Furthermore, when Big Data are missing key covariates, it may be difficult to account for the effect of confounding factors (sex, socioeconomic determinants, ethnicity). An additional important challenge concerns the assessment of the mental health outcome itself. While the development of advanced sentiment analysis function as a proxy for highlighting emotional distress in the digital sphere, this type of approach precludes any formal assessment of actual mental health outcomes and may result in distorted conclusions. Big Data is also prone to p-hacking (manipulation of data to achieve statistical significance) and harking (hypothesizing after the results are known), especially if the data contains many variables. Hence, a pre-registered analysis plan adds credibility. This plan should include an adjusted significance level, because very small effects may become significant by chance when working with Big Data. Finally, claims of causality cannot be made; therefore, data have to be interpreted carefully. Overall, the strict adherence to reporting guidelines is of utmost importance to overcome methodological concerns.

## Strengths of Big Data

Despite these concerns, Big Data analysis may contribute to a more comprehensive understanding of the mental health consequences from the current COVID-19 crisis. Big Data are not only “long” (covering many individuals), they are also “large”, that is, they contain many variables that are already included or that can be easily extracted from these data [6]. The main strength of this approach, however, is the huge data volume made available even across national borders and health care systems. Thereby, dozens of millions of e.g., geo-referenced Twitter tweets, may be analyzed, substantially increasing the statistical power of spatial analyses linking mental health determinants, COVID-19 case counts or regulations, and sentiments of social media users in those locations [10]. Therefore, Big Data analyses could help identify regional differences and establish correlations with other factors such as incidence rates of COVID-19, lockdown strictness or other policies aimed at containing the pandemic, or hospital overcrowding. Analysis of big social media data in combination with spatial epidemiological approaches may further identify geographic hotspots of increased symptoms of mental health problems over time [7]. This in turn could provide key operational information to help implement appropriate mental health support and prevention measures. Moreover, real time monitoring of the mental health consequences of COVID-19 may help set up governments to respond rapidly and appropriately to changes in mental health status. Unlike formal epidemiological studies, the huge data volume and wide geographic coverage of Big Data surveillance come at limited costs and in real-time, making this approach an efficient use of resources. The main limitations are computational power, interpretability, and threats to generalizability.

## Conclusion

We recommend the use of Big Data approaches to monitor mental health in the general population, especially in the context of heightened anxieties and threats to mental wellbeing owing to the COVID-19 pandemic, as there may be ways to leverage these novel data sources to help deliver targeted support to specific populations including those who are most susceptible to the impacts of the pandemic and resulting mental health consequences. Hence, Big Data hold potential to strengthen our mental health prevention systems in the context of a global public health crisis. There will be ethical and technical challenges, which will require careful and continued efforts to overcome, but these digital approaches can support multifaceted strategies including both modern technologies and traditional approaches.
